# Surgical management of the congenital dislocation of the knee and hip in children presented after six months of age

**DOI:** 10.1007/s00264-020-04759-8

**Published:** 2020-08-08

**Authors:** Amrath Raj B.K., Kumar Amerendra Singh, Hitesh Shah

**Affiliations:** Department of Orthopaedics, Kasturba Hospital, Kasturba Medical College, Manipal Academy of Higher Education, Manipal, 576104 India

**Keywords:** Reduction, Spontaneous, Congenital dislocation of the hip, Congenital dislocation of the knee

## Abstract

**Purpose:**

Congenital dislocation of the knee and hip is a rare congenital disorder. The specific aim of the study was to evaluate the clinical and radiological outcomes of the children with congenital dislocation of the knee and hip who presented after six months of age.

**Methods:**

All the consecutive children with congenital dislocation of the knee and hip joints were retrospectively reviewed. We included cases that were treated after six months of age and followed up for a minimum of two years. Twenty-four children with congenital dislocation of the knee and hip (thirteen with ligamentous laxity, eleven children with stiff joints) were included. The knee was dislocated in 45 limbs; the hip was dislocated in 40 instances. The knee joint dislocation was treated with quadricepsplasty in all twenty-four children (45 knees). The hip dislocation (*n* = 32) was addressed with either closed reduction (*n* = 8) or open reduction of the hip (*n* = 24). Eight hip dislocations were not addressed. The outcome of the hip and knee was evaluated.

**Results:**

The clinical and radiological outcomes were better in children with ligamentous laxity than without laxity. Twenty-two children were community walkers. An orthosis was needed in eight children. The frequency of spontaneous reduction of unreduced dislocation of the hip was noted in three children (5/8 hips).

**Conclusion:**

Outcome in combined dislocation of knee and hip is good in most cases with surgical interventions. The outcome is better in children with ligamentous laxity. Spontaneous reduction of the dislocated hips might be achieved after gaining knee flexion following knee surgery for congenital the knee in a few cases.

**Electronic supplementary material:**

The online version of this article (10.1007/s00264-020-04759-8) contains supplementary material, which is available to authorized users.

## Introduction

Congenital dislocation of the knee (CDK) is a rare congenital disorder. A congenital dislocation of hip (CDH) is common with congenital dislocation of the knee. Association of the CDK and CDH are common in various pathologies like Larsen syndrome, arthrogryposis multiplex congenita, sacral agenesis, and diastrophic dysplasia [1].

Younger infants can be treated with serial manipulation and casting for CDK. Once 90° of flexion of the knee joint is achieved, Pavlik harness is indicated. Infants with presentation beyond six months of age, surgical intervention is warranted [2]. Children with failed conservative treatment can also be treated with the surgical intervention [1].

There is a lack of literature regarding the timing, sequence, and type of reduction in children present around walking age. It is unusual to have a late presentation beyond six months. However, the reasons for late presentation are failure of conservative treatment, a poor socioeconomical background of parents, and late referral to the tertiary centre. Few recommended surgical reduction of knee first followed by hip, while others suggested simultaneous correction of the hip and knee with femur shortening. However, before addressing the associated hip dislocation, one must get 90° of knee flexion to relax hamstrings [3].

The specific aims of the study were to evaluate the clinical and radiological outcomes of the children with CDK and CDH who presented after 6 months of age and compare the outcome between children with ligamentous laxity and without ligamentous laxity.

## Material and methods

A retrospective study was planned, and ethical committee clearance was taken for the same. All 40 consecutive children with combined CDK and CDH who have taken treatment in this tertiary care institute from 2005 to 2016 were retrospectively reviewed. Children presented early (less than 6 months) were treated with serial manipulation and casting. Children presented late, or failure of conservative treatment was treated with quadricepsplasty. We included only those children treated with quadricepsplasty.

The inclusion criteria were (1) children with combined CDK and CDH, (2) children who were treated with quadricepsplasty (either presentation after 6 months or failed conservative treatment), and (3) a minimum two year follow-up after the index surgery. Children with isolated CDK, isolated CDH, a child treated with conservative treatment, less than two years follow-up, and spinal dysraphism were excluded. Sixteen children were excluded (three children with spinal dysraphism, nine children treated with serial manipulation and cast, four children with inadequate follow-up).

Twenty-four children (15 girls, nine boys) formed the basis of the study. Among 24 children, 16 were late presented, while eight had failed conservative treatment. The clinical data were retrieved from medical records. Arthrogryposis is widely recognized as “a child born with contractures of two or more body regions”. Hence, all children included in this study had arthrogryposis multiplex congenita [4]. However, there was a clear pattern of ligamentous laxity in a few children, while a few children were stiff. Four or higher Beighton score was considered as a child with ligamentous laxity [5]. Children with and without ligamentous laxity were grouped separately. Thirteen children had a ligamentous laxity, while eleven children had stiff joints. All serial radiographs were studied from the first presentation to the latest follow-up. The knee was dislocated (complete dislocation of femoral tibial joint) in 45 instances; the hip was dislocated in 40. The club foot was present in 14 feet, while 10 feet had associated congenital vertical talus (CVT). The demographic data in both groups were comparable (Table 1).

**Table 1:** Demographic data of all children with congenital dislocation of knee and congenital dislocation of the hip

Variables		Total	Children with ligamentous laxity (*N* = 13)*	Children without ligamentous laxity (*N* = 11)*	*p*
The age at presentation (months) (Mean + SD)		7.95 + 2.40	7.92 + 2.06	8.27 + 2.90	0.734
Gender (number of children)	Boy	9	5	4	0.916
	Girl	15	8	7	
Knee dislocation (*n* = 45)		45	23	22	
Side (number of children)	Unilateral	3	3; R (1), L (2)	0	0.089
	Bilateral	21	10	11	
Hip dysplasia (*n* = 40)		40	23	17	0.005
Side (number of children)	Unilateral	8	3; R (1), L (2)	5; R (3), L (2)	0.292
	Bilateral	16	10	6	
Tonnis	II	23	15	8	0.404
	III	10	4	6	
	IV	7	4	3	
IHDI	II	12	10	2	0.046
	III	16	9	7	
	IV	12	4	8	
Foot deformity (number of feet)	Equinovarus	14	2	12	< 0.001
	CVT	10	1	9	
	Normal	21	20	1	
The age at surgery of the hip joints (months) (Mean + SD)		22.37 + 13.58	16.58 + 10.71	28.93 + 13.81	0.008
The age at the final follow-up (months) (Mean + SD)		87.00 + 41.19	69.07 + 34.72	106.68 + 38.34	0.019

*N number of children; *n* = number of limbs; *CVT* congenital vertical talus

### Treatment protocol

Forty-five knee joints (24 children) were treated by modified Curtis and Fischer quadricepsplasty [6, 7]. Three modifications to the original technique were lateral incision instead of the midline, transverse division of rectus femoris at the musculotendinous junction, and preservation of both side retinacula and collateral ligaments. After gaining 90° knee flexion, the knee was immobilized in 90° flexion for four weeks. Children with bilateral dislocation of the knee were operated simultaneously. Thereafter, supervised physiotherapy was done for six weeks.

Once the knee dislocation was reduced, associated foot deformity was corrected with soft tissue release (14 clubfeet, 10 CVT) after three months. Twelve clubfeet and 9 CVT were with stiffness, whereas only two children with ligamentous laxity had foot deformity (2 clubfeet, 1 CVT). The feet were immobilized for six weeks. After immobilization, the correction was maintained with ankle-foot orthosis.

A reduction of the dislocated hip (32 hips) was done after three months following foot surgery. Surgical intervention for the hip dislocation included closed reduction (8 hips), open reduction (17 hips), and open reduction with femur osteotomy (7 hips) depending on the age. All surgeries were done through the anterolateral Somerville approach. The reduced hip was maintained in a spica for three months. Fixed abduction-splint was used for an additional three months in children with laxity. The stiff hip dislocation was mobilized after three months. No intervention was done in eight hips (4 children; one with stiffness, three with laxity).

Three stiff hips (3 children) and five hips (4 children) with ligamentous laxity required spica after closed reduction. Open reduction and spica application were done for six stiff hips (5 children) and 11 lax hips (7 children). A femur shortening with the open reduction was required in nine stiff hips (5 children) and only one child with ligamentous laxity.

All these children were treated and followed up clinically and radiologically at regular intervals. The mean age at quadricepsplasty was 7.95 months (6–16 months). The mean duration of follow-up was 87 months (24–181 months).

### Clinical assessment

All children were evaluated for activity level, pain, limp, weakness, and use of a brace. The presence of deformity and the limb-length discrepancy was noted. The passive range of movement of the knee joint at the final follow-up was compared with the pre-operative range. The presence of extensor lag, degree of hyperextension, and flexion deformity of the knee was noted. The power of quadriceps and hip abductors was graded on the Medical Research Council (MRC) scale. The stability of the knee joint was checked in sagittal and coronal planes. All the hip joints were rated using the McKay criteria [8].

### Radiological assessment

The pre-operative radiographs were studied for the severity of knee dislocation, Tonnis grades [9], and International Hip Dislocation Institute (IHDI) grades [10]. All the follow-up radiographs were evaluated for the development of femoral head, femoral neck, acetabulum, and hip joint congruity. The children older than six years were categorized into different Severin classes [11].

### Statistical methods

A chi-square test was used to compare the frequency distributions between both groups. The independent *t* test was used to compare continuous variables. The clinical and radiological outcomes at the final follow-up were compared between these two groups.

## Results

**Fig. 1:**
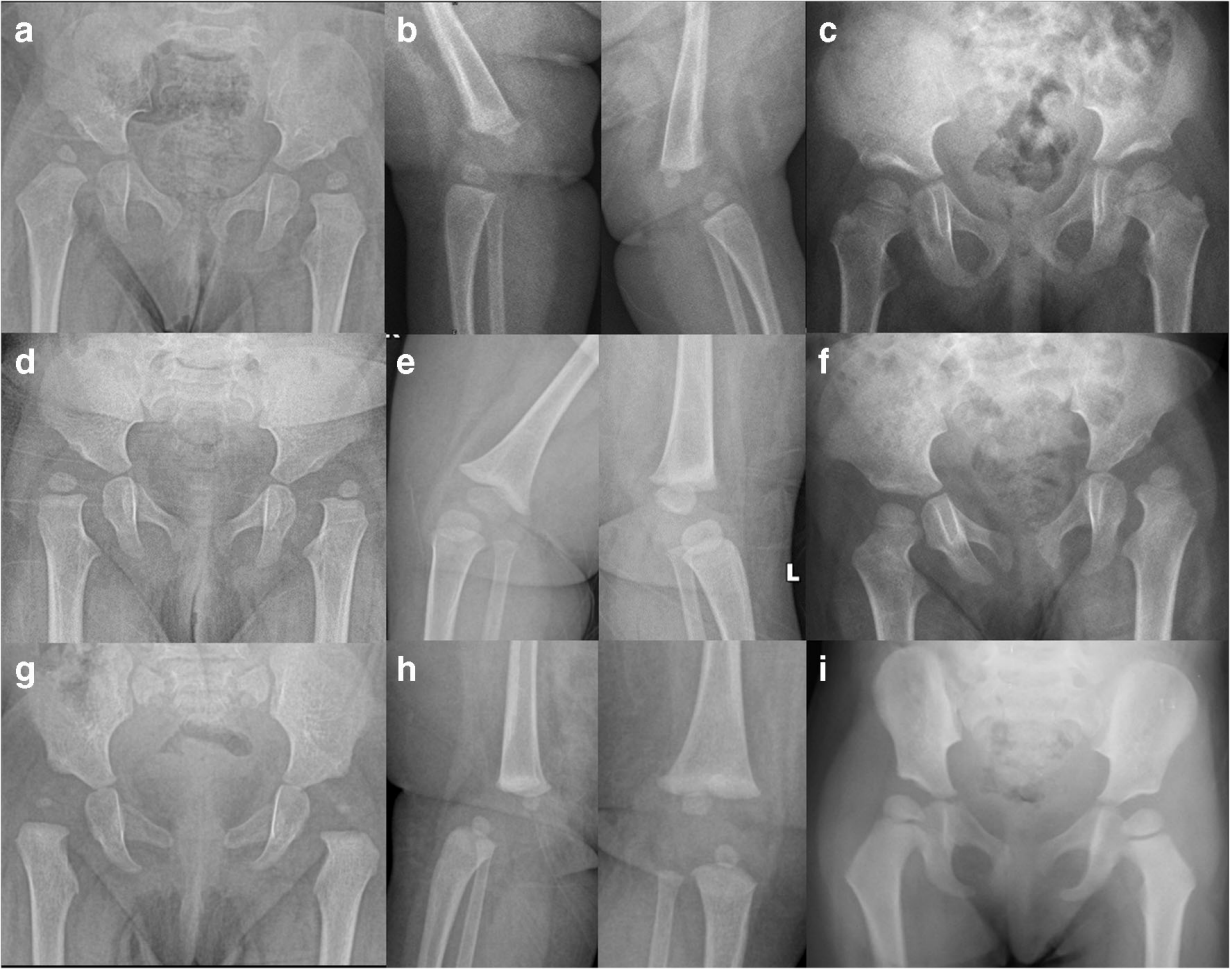
Nine-month girl (case 1) brought with bilateral congenital dislocation of the knee and bilateral CDH (**a**, **b**). She underwent bilateral quadricepsplasty. Radiograph taken at 3.5 years of age showed a spontaneous reduction of both the hip joints (**c**). Another 9-month girl (case 2) brought with bilateral congenital dislocation of the knee and CDH (**d**, **e**). She underwent bilateral quadricepsplasty. Radiograph at 15 months follow-up showed a spontaneous reduction of the right hip joint (**f**). The 6-month girl (case 3) presented with bilateral CDH and CDK (**g**, **h**). She underwent bilateral quadricepsplasty. Radiograph at 2-year follow-up showed a spontaneous reduction of both the hip joints (**i**)

The mean age at the first surgery was 7.95 months. The mean age at final follow-up was 87 months. All the children without ligamentous laxity had bilateral involvement. Twenty-one limbs (95.45 %) without laxity had foot involvement. Three children (23.07 %) in the ligamentous laxity group had single limb involvement, and only three limbs (11.53 %) had foot involvement. Spontaneous reduction of the hip joint was noted in three children (5 hips) (Fig. 1).

### Case 1

A nine month girl, first born, presented with bilateral CDK, CDH, and ligamentous laxity. Both knee and hip dislocations were not reducible. Radiographs confirmed bilateral Tonnis II hip and knee dislocations. Bilateral single-stage modified quadricepsplasty improved knee flexion to 120°. She was lost in follow-up after six weeks. She presented at the age of two years (1 year after the index surgery) with spontaneous reduction of the bilateral hips. She did not take any treatment after the first surgery (operative or non-operative). Her hips and knee were stable with an excellent range of motion. The acetabulum remodeling is also noted on both sides at 3.5 years of age (Fig. 1a–c).

### Case 2

A nine month girl, the second-born, presented with bilateral CDK and Tonnis II CDH. Her knee and hip dislocations were not reducible. Single-stage bilateral quadricepsplasty improved knee flexion to 90°. Her parents refused for the treatment of dislocated hips. At 15 months, follow-up x-rays revealed a spontaneous reduction of the right hip with the remodeling of right acetabulum, while the left hip remained dislocated (Fig. 1d–f).

### Case 3

A six month girl presented with bilateral CDK, CDH, and ligamentous laxity. Closed reduction of both the knee and hip dislocation was not possible. Single-stage bilateral quadricepsplasty improved both knee flexions to 110°. She was planned for surgical correction of dislocated hips. However, both hips were found to be spontaneously reduced at follow-up. Hence, no further intervention was done (Fig. 1 g–i).

### Range of movement and stability, muscle power

The mean passive flexion of knee joint improved to 101° at the final follow-up. The mean passive flexion of the knee joint noted in the laxity group and without the laxity group was 113° and 88°, respectively (*p* < 0.001). The children without laxity had more severe flexion deformity when compared with the children with laxity (*p* < 0.05). Three children showed grade I anterior instability in the laxity group. Two children demonstrated medial instability. No child with stiffness had knee joint instability. The median quadriceps power was 4.

**Fig. 2:**
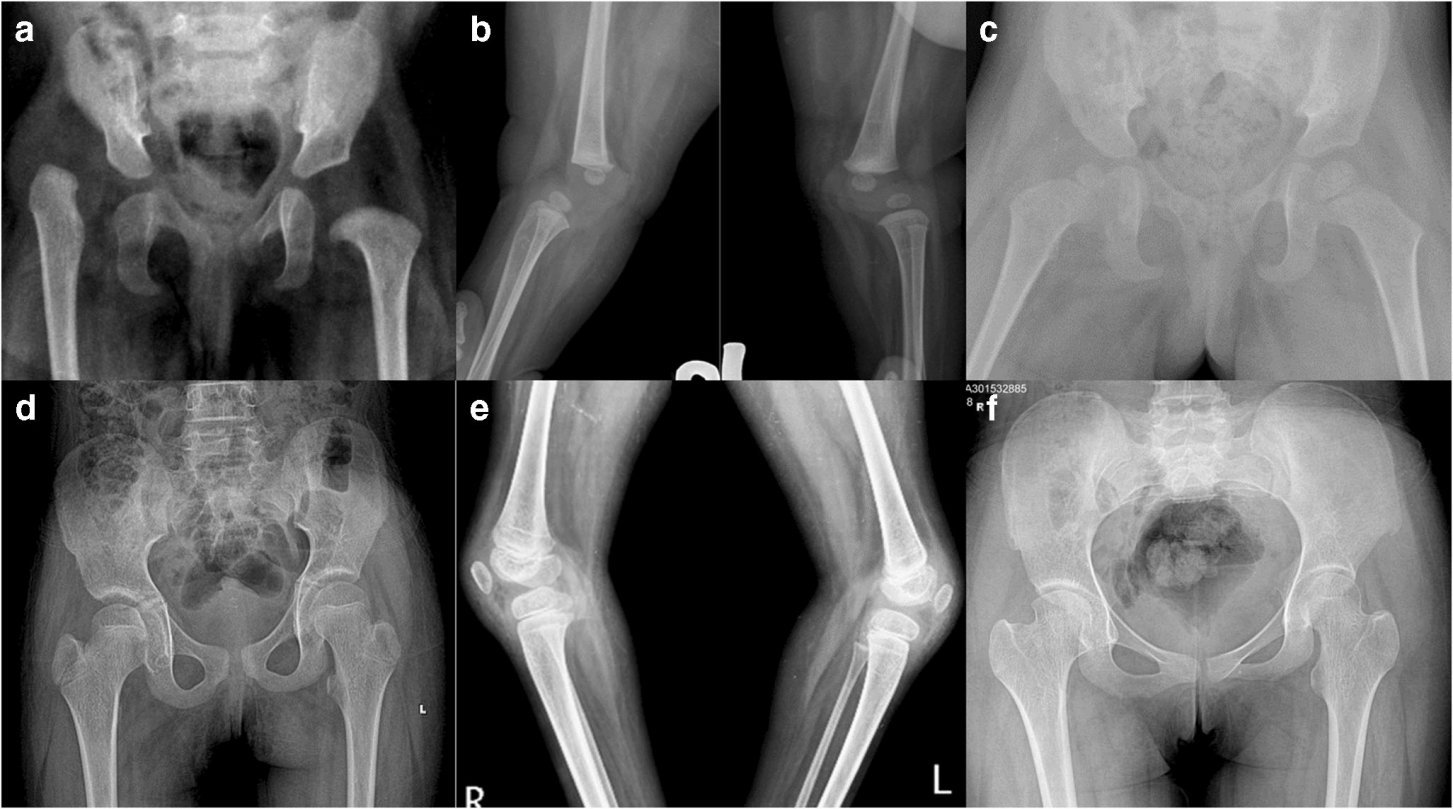
Serial radiographs of a child with arthrogryposis (**a**, **b**) who was operated at seven months for knee, and hip dislocation was reduced with open reduction at 2 years of age. Right acetabular dysplasia was persistent at six months from surgery (**c**). Excellent alignment of hip and knees at 14 years (**d**, **e**). She showed a good outcome of the hip at skeletal maturity with the remodeling of the right acetabulum (**f**).

**Fig. 3:**
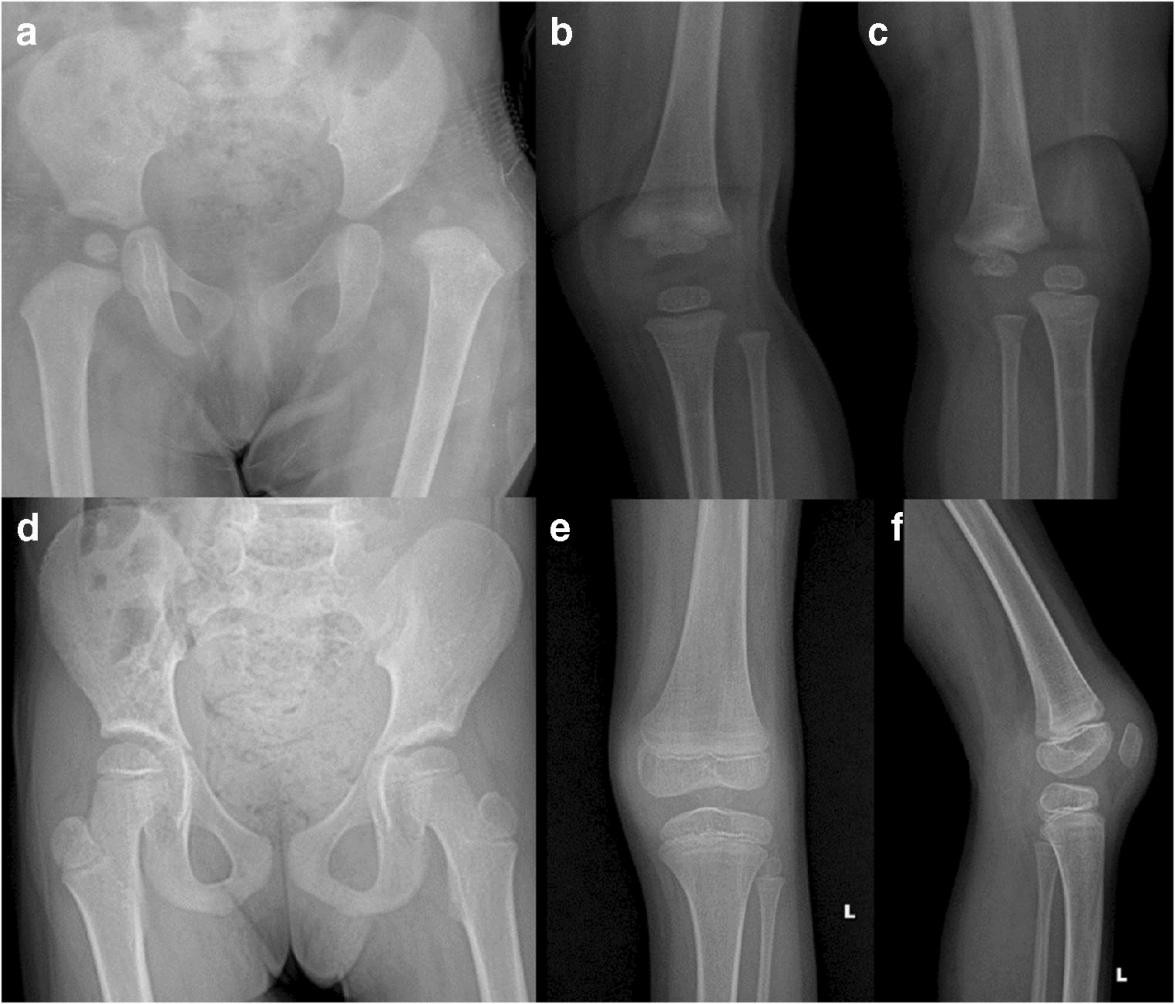
One-year girl presented with left hip and knee dislocation (**a**–**c**). She missed any features suggestive of arthrogryposis. A closed reduction of left hip followed left quadricepsplasty. She has a good outcome of hip and knee joints at 69-month follow-up (**d**–**f**)

McKay rating showed excellent results in nine hip joints. The rating was better in children with ligamentous laxity. The radiological parameter measurements were reproducible (intra-observer and inter-observer). Severin classification [11] was good in 67% of children. The radiological outcome was similar in both groups (Figs. 2 and 3). Three hip joints in children without joint laxity had subluxation. Coxa magna was seen in five hips (3 with laxity, two without laxity).

### Functional evaluation

**Fig. 4:**
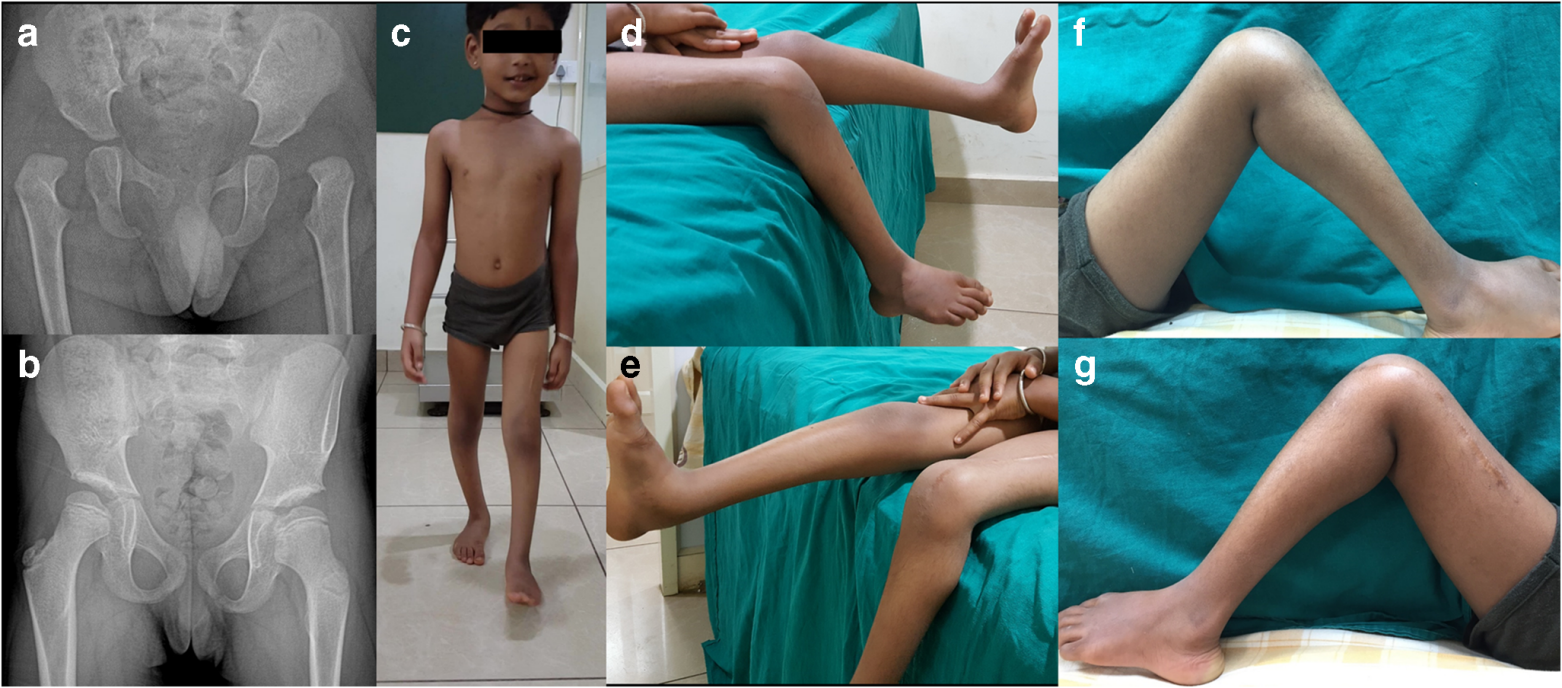
Clinical photographs and final radiographs of a child with ligamentous laxity showed good results

All except one child were community walkers in children with ligamentous laxity group (Fig. 4). One child was a household walker. The same child used braces for walking. Among the child without laxity, ten were community walkers. Seven children required braces for their ambulation (Table 2).

**Table 2:** Outcome of children with congenital dislocation of the knee and congenital dislocation of the hip

Variables		Total (*N* = 24)*	Children with ligamentous laxity (*N* = 13)*	Children without ligamentous laxity (*N* = 11)*	*p*
Outcome of the knee joint	Final knee flexion (degrees) (Mean + SD)	101.33 + 17.26	113.91 + 4.99	88.18 + 15.62	< 0.001
	Extensor lag of the knee joint (degrees) (Mean + SD)	4.00 + 4.07	2.83 + 3.93	5.23 + 3.92	0.047
	Fixed flexion deformity of the knee (degrees) (Mean + SD)	0.67 + 1.72	0	1.36 + 2.27	0.011
	Quadriceps power (MRC grade) (median)	4	4	4	0.157
Outcome of the hip joints	McKay ratings (number of hip joints)	Excellent-9 Good-16 Fair-13 Poor-2	Excellent-9 Good-12 Fair-2 Poor-0	Excellent-0 Good-4 Fair-11 Poor-2	< 0.001
	Severin classes (number of hip joints)	Class I-8 Class II-6 Class III-4 Class Iva-1 Class IVb-2	Class I-2 Class II-3 Class III-2 Class IVa-0 Class IVb-0	Class I-6 Class II-3 Class III-2 Class IVa-1 Class IVb-2	0.558
	Center-edge angle (degrees) (Mean + SD)	13.23 + 12.26	15.46 + 7.74 (7 hips)	12.11 + 14.13 (14 hips)	0.569
Ambulation	Orthosis needed for ambulation (*n* = 18 limbs)	8 children	1 child 2 limbs	7 children 14 limbs	< 0.001
	Ambulatory status (*n* = 25)	24 children	Community-12 Household-1	Community-10 Household-1	0.902

N* number of children

### Secondary surgeries and complications

**Fig. 5:**
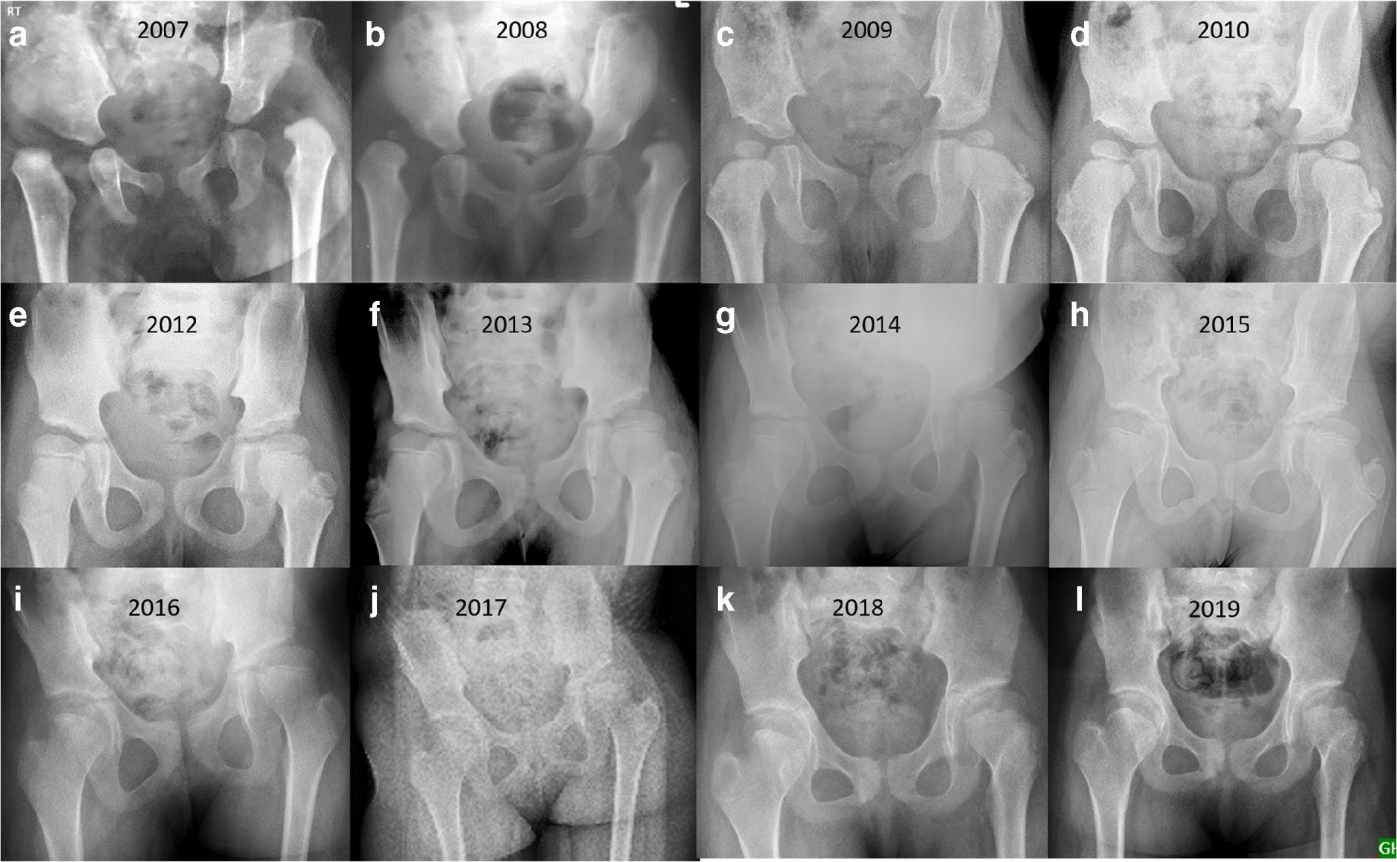
Serial radiographs of a child without ligamentous laxity showed good outcomes 12 years after surgery (**a**–**l**). Lateral growth arrest of the proximal femur physis was present with the excellent remodeling of the acetabulum

Secondary corrective surgery was required in four children. These surgeries were varus derotation osteotomy (*n* = 3) and acetabuloplasty (*n* = 1). Two children without laxity showed persistent subluxation. Avascular necrosis was noted in four hips (type I in 2, type II in 2; 4 children) (Fig. 5). No severe deformity was noted in any child. The frequency of secondary procedures and avascular necrosis were the same in both groups. One child developed a proximal femur fracture two years following hip surgery. The fracture was treated with osteosynthesis. The hip was subluxated at the last follow-up.

## Discussion

Combined congenital dislocation of the knee and dislocation of the hip is rare. There are two different approaches to the reduction of ipsilateral knee dislocation and hip dislocation. Traditionally, the knee is reduced first, followed by a reduction of the hip joint [12]; however, Johnston CE showed good results with simultaneous reduction of the hip and knee joint with femur shortening [1].

### The outcome of the knee dislocation

Johnston CE defined the indication of the soft tissue lengthening for the treatment of the congenital dislocation of the knee [1] Different approaches to correct the congenital dislocation of the knee include minimal invasive technique, percutaneous surgery, needle tenotomy, traditional open lengthening of the quadriceps muscle [6], or modification of the quadricepsplasty [7].

### The outcome of the hip dislocation

There is no consensus in the literature regarding addressing the hip dislocation in nonsyndromic, syndromic, and arthrogryposis. The child with bilateral dislocation was left alone in Larsen syndrome and arthrogryposis [3]. However, recent trends showed good results with the early intervention for the hip dislocation in the child with stiffness [13, 14].

The closed reduction was adequate in cases with ligamentous laxity. However, closed reduction is rarely successful in arthrogryposis children [13] and Tonnis grade IV [15, 16]. These hips invariably require open reduction or open reduction with femur osteotomy. Few authors described good results with a medial open reduction [17], while others described similar results with the anterolateral approach [18]. Restricted range of motion of the hip was noted with the treatment of the hip dislocation [13].

In this study, most hips were addressed with closed reduction, open reduction, and open reduction with femur osteotomy. The children with stiffness required open reduction or open reduction with femur osteotomy. Children with ligamentous laxity were treated with closed reduction. The spontaneous reduction of the hip was noted in five hip joints in three children with laxity.

### Functional outcome of the children

The ambulatory capacity of the syndromic, nonsyndromic, or arthrogryposis children depends on the associated deformities. The children with knee flexion contracture and hip dislocation were associated with nonambulatory status in arthrogryposis [19]. The early intervention with aggressive physiotherapy showed good function in a child with arthrogryposis [14]. An orthosis was needed in a few cases with foot deformities. Most children in the present study had community ambulation. The functional status was better in children with laxity.

### Compare with other studies

We have compared our results with similar studies in the literature (Table 3). To the best of our knowledge, spontaneous reduction of the dislocated hips after walking age has not been reported in the literature.

**Table 3:** Comparison of the results of congenital dislocation of the knee and hips with other studies

Sl.no	Authors	Year	N*	Outcome		Other deformities	Complications	Comment
				Hip joint *N* (*n*)	Knee joint *N* (*n*)			
1	Curtis BH et al.[6]	1969	11	11 Outcome not mentioned	11 (15) Good:7, Fair:3, Poor:2	CTEV-7 Calcaneovalgus-1	One knee joint required arthrodesis	AMC-7, Lateral placement of the patella > 50%
2	Iwaya T et al.[20]	1983	3	3 (4) Good:2, Unreduced:2	3 (3) Excellent:2, Good:1	CTEV-1	Both hips failed to reduce in a child with cerebral palsy	Cerebral palsy-1
3	Jacobsen K et al[21]	1985	8	8 (12) Outcome not mentioned	8 (13) Outcome not mentioned	CTEV-10 Spinal anomalies-3		Down syndrome-1, Larsen syndrome-1, MMC-1, AMC-1
4	Ferris B et al.[2]	1987	4	4 (6) Outcome not mentioned	4 (6) Excellent-4, Good-1, Fair-1	CTEV-2 Syndactyly-1		
5	Oetgen M et al[22]	2010	5	5 (8) Outcome not mentioned	5 (8) Knee flexion 122 degrees	CTEV-6 Spinal anomalies-2		Larsen syndrome in 5/7 children.
6	Cheng C et al[23]	2010	14	14 (22) All - Pavlik harness. 2 required an acetabular osteotomy	14 (19) Excellent-17, Good -1	CTEV-4 Hind-foot valgus-2	One child with bilateral DDH, CTEV, CDK and corpus callosum agenesis died	AMC-1
7	Roth S et al [24]	2010	3	3 (6) Pavlik harness. All joints good outcome	3 (6) Good- 3, Poor-3			All three children were delivered prematurely. One knee operated
8	Abdelaziz T et al[3]	2011	7	7 (13) Outcome not mentioned	7 (13) Excellent-6, Good-5, Fair-2	CTEV-2 Calcaneo-valgus-4	Deep infection-3, Recurrent dislocation-3 Recurrent Genu valgum-2	All knees were operated
9	Johnston CE[1]	2011	8	8 (11) Severin:1A-1, 1B-1,2A-5 2B-1, 3-2, 4-1	8 (11) Excellent-3, Good-1, Fair-3, poor-4		Tibial growth arrest-1 Repeat hip open reduction-1 Additional hip surgery-2	Larsen syndrome-4 Idiopathic-2 Trisomy8-1 Diastrophic dislocation-1
10	Tercier S et al[7]	2012	17	17 All children became community walker	17 All children became community walker			Better results in nonsyndromic cases
11	Current study	2020	24	24 (40) McKay rating Excellent-9, Good-16 Fair-13, Poor-2	24 (45) Passive knee flexion 101.33 degree, Mean knee extensor lag -4 degree	CTEV-14, CVT-10	FFD knee-0.67 , Three hip joints subluxated at final follow-up	Spontaneous reduction 5/8 hip joints

*N* Number of children, *n* number of joints, *CTEV* congenital talipes equinovarus, *CVT* congenital vertical talus, *AMC* arthrogryposis multiplex congenita, *MMC* meningomyelocele

### Spontaneous reduction of the hip

Recently, treatment of CDH by closed reduction and short leg cast showed excellent outcomes in Tonnis grade II and III [16]. Bilateral dislocation and Tonnis grade IV is associated with the failure of the reduction of the hip joint. Similarly, closed reduction and dynamic cast were also described to maintain reduction [25]. Szepesi et al. [26] mentioned good long-term results with open reduction and early mobilization without spica cast.

All three children with spontaneous reduction of the hip had gross ligamentous laxity. All spontaneously reduced hips were Tonnis grade II/III. None of Tonnis grade IV hips showed a spontaneous reduction. These hip and knee joints were not reducible under anaesthesia. The dislocation of the knee required quadricepsplasty for reduction. They did not take any treatment for hip dislocation. The spontaneous reduction of the hip might be attributed to the relaxed hamstring. Persistent quadriceps contracture with dislocation of the knee leads to severe hamstring stretching. Once the knee flexion is achieved, hamstrings are relaxed. Stretched hamstring might play an important role in the dislocation of the hip joint. It can contribute to secondary hip dislocation. Following knee surgery, all children were immobilized in 90° of knee flexion. With knee flexion, hip might be flexed or abducted. These might help to relax the adductors and flexors of the hip joint. Hence, it might be the reason to achieve a spontaneous reduction of the hip. However, we did not get a spontaneous reduction in all dislocated hips. Four children (8 hips) were not treated for hip joint dislocation. Three hips remain dislocated at the final follow-up. The surgery is being planned to reduce the hip joint dislocation.

### Limitation of the study

This study is a retrospective study. All children have not attained skeletal maturity. We intend to follow up on these children to skeletal maturity. The study includes a diverse population ranging from arthrogryposis multiplex congenita, hyper-laxity syndrome, and nonsyndromic children. Hence, the functional scoring would be different in stiff and lax joints. The five hips were normal, associated with CDK. However, the unilateral CDH was included with bilateral dislocation of the knee joints. The number of children in each group is less, so it is difficult to draw a robust, meaningful conclusion of the approach and spontaneous reduction of the hip. A future multicentric prospective study of such a rare congenital disorder will answer the exact outcome in both groups.

### The uniqueness of the study

The study includes the largest case series of combined knee dislocation and hip dislocation who presented after six months of the age. The spontaneous reduction of the hip could be achieved after the reduction of the knee joint even after nine months of age in children with hyper-laxity. The outcome of the knee and hip surgeries would be different in the child with stiffness and the child with laxity.

## Conclusion

The outcome in combined CDK and CDH is good in most cases with surgical interventions. The result is better in children with ligamentous laxity. Spontaneous reduction of the dislocated hips might be achieved after gaining knee flexion following knee surgery for congenital dislocation of the knee in a few cases.

## Electronic supplementary material

ESM 1 (XLSX 320 kb)
